# Detection and classification of venous thromboembolism through image test reports analysis using active learning and deep learning

**DOI:** 10.1371/journal.pone.0335262

**Published:** 2025-11-10

**Authors:** Eunseon Jeong, Youngje Woo, Hong Joo Lee, Jang Yong Kim

**Affiliations:** 1 Department of Surgery, Division of Vascular and Transplant Surgery, Seoul St. Mary’s Hospital, The Catholic University of Korea College of Medicine, Seoul, Republic of Korea; 2 Department of Healthcare & Artificial Intelligence, The Catholic University of Korea, Bucheon, Gyounggi-do, Republic of Korea; Faculty of Medicine of Alexandria University: Alexandria University Faculty of Medicine, EGYPT

## Abstract

**Objective:**

This study evaluated whether active learning can enable efficient classification of venous thromboembolism (VTE) reports using minimal labeled data. In parallel, we assessed whether deep learning (DL) models can achieve substantially superior performance compared to traditional machine learning (ML) models and overcome the limitations associated with small sample sizes and class imbalance in real-world clinical datasets.

**Methods:**

5,839 imaging reports with, of which 1,088 (18.6%) were VTE-positive. Traditional ML models (RF, SVM, SVM with SGD, GBM) were combined with active learning strategies (random sampling, uncertainty-based, word similarity, TF-IDF similarity). DL models (LSTM, multi-kernel 1D-CNN with GloVe, BERT-based models) were also evaluated. F1 scores were used as the performance metric.

**Results:**

Among VTE-positive patients, 65.0% had corresponding ICD-10 codes, indicating frequent under-documentation. ML models with active learning achieved F1 scores of 0.70–0.80, while DL models, particularly LSTM and multi-kernel 1D-CNN with GloVe achieved F1 scores ≥0.94 in a 7-class classification, even under severe class imbalance. Excluding the “No DVT and PE” class for a 6-class classification among VTE-positive cases led to reduced model performance, with the largest decline observed in BioBERT. The average inference time per report ranged from 0.0014 to 0.024 seconds depending on the model architecture, suggesting that the system is feasible for near real-time deployment in clinical settings.

**Conclusion:**

DL models substantially outperformed traditional ML in classifying VTE reports, with high accuracy, acceptable inference time, and robustness to class imbalance. These models hold promise for augmenting clinical workflows, particularly in addressing under-coded but clinically significant VTE cases.

## Introduction

Deep vein thrombosis (DVT) is a type of venous thromboembolism (VTE) in which a blood clot forms in the deep veins of the lower leg or thigh. Although DVT is often asymptomatic, severe DVT can markedly impede venous return in the lower extremities, potentially causing swelling or pain. In some cases, the clot dislodges from its original site, traveling to the pulmonary artery (pulmonary artery thromboembolism) or passing through a patent foramen ovale into systemic circulation, leading to paradoxical embolism [1]. In the United States, VTE is recognized as a leading cause of preventable cardiovascular disease, a major postoperative complication, and the number one preventable cause of death [2]. Each year, 375,000–425,000 VTE cases occur post-surgery, incurring per-case costs of $12,000 to $15,000 [3]. Furthermore, pulmonary embolism is an acutely life-threatening post-operative complication, and chronic venous thromboembolism can lead to pulmonary arterial hypertension and associated right heart failure [4].

The main pathophysiology of VTE is described by Virchow’s triad (stasis of blood flow, epithelial injury of the vessel, and a hypercoagulable state of the blood). Various risk factors are implicated, such as immobilization due to trauma or surgery, malignancy, inflammatory bowel disease, and autoimmune diseases [5]. Hospitalized patients, in particular, can be considered a high-risk group for VTE because they often suffer from acute or chronic illnesses with multiple overlapping risk factors [6]. For inpatients deemed to be high risk for VTE, the most crucial step is appropriate VTE prophylaxis such as anticoagulants, compression stockings, intermittent pneumatic compression (IPC), or early ambulation depending on each patient’s individualized risk [7,8].

Analyzing ICD-10 codes has limitations for accurately identifying VTE prevalence because actual inpatient diagnoses are often omitted. VTE that occurs secondarily to other conditions might not be systematically coded, and discrepancies can occur between imaging test reports and discharge diagnoses [9]. Although some algorithms incorporating treatment data and imaging results have been proposed to improve VTE detection sensitivity, issues of insufficient sensitivity and validity in large-scale datasets remain problematic [10–14].

An alternative approach is to analyze the original radiologic test reports instead of ICD-10 codes, potentially yielding a more accurate estimate of VTE prevalence. However, those reports contain free-text descriptions and complex clinical information unique to each patient, so attaining high accuracy with simple rule-based methods is difficult. Machine learning can identify significant patterns in large text datasets; models such as the Support Vector Machine (SVM), Random Forest (RF), and Gradient Boosting Machine (GBM) each handle high-dimensional data but in different ways. Combining machine learning with active learning can substantially reduce labeling costs, because the most uncertain or information-poor data can be prioritized for annotation [15–17].

Nevertheless, machine learning alone might be insufficient to fully capture the contextual relationships within unstructured, complex text. Therefore, deep learning models have garnered attention. For instance, BERT (Bidirectional Encoder Representations from Transformers) learns bidirectional context via a self-attention mechanism to improve understanding of word interactions within sentences [18,19]. BioBERT and BioLinkBERT further incorporate biomedical domain texts or knowledge graphs to enhance model accuracy in medical applications [20,21]. As other deep learning options, one-dimensional convolutional neural networks (1D-CNNs) can quickly capture local text patterns, while Long Short-Term Memory (LSTM) models excel at maintaining context over long sequences [22,23].

In this study, we compare the performance of machine learning models (SVM, RF, GBM) for classifying VTE-related test reports with that of deep learning architectures (BERT-based models, 1D-CNN, LSTM). We added active learning to the machine learning framework to investigate whether high accuracy could be achieved with minimal labeling. Our goal for this exploration was to identify the most appropriate technique for VTE report classification and lay the groundwork for an automated model that could be practically used in clinical environments.

## Materials and methods

### Datasets of interest

The dataset used in this study comprises imaging test reports from inpatients suspected of having VTE at The Catholic University of Korea Seoul St. Mary’s Hospital from May 1^st^ 2016 to May 31^st^ 2022. The tests we analyzed were chest computed tomography (CT), pulmonary artery CT angiography, DVT CT, lower extremity vein CT, and lower extremity vein duplex ultrasonography. Additionally, demographic factors (patient age and sex) and ICD-10–based diagnoses were collected. The hospital’s Information Strategy Team extracted the relevant data, and all personally identifiable information including hospital identification codes and names was anonymized. Authors could have accessed the extracted data since July 3^rd^ 2023, and the authors did not have access to information that could identify individual participants during after data collection.F1.

A skilled expert examined each test report in the collected dataset to determine the presence and anatomical location of pulmonary embolism (PE) and DVT. PE was defined as the presence of a thrombus in the truncal, segmental, or subsegmental region of the pulmonary artery. DVT was classified according to its location: proximal (from the inferior vena cava to the popliteal vein), distal (soleal and calf veins), thrombophlebitis (great or small saphenous veins), and others (thrombi in the subclavian vein, internal jugular vein, upper extremity veins, etc.).

According to those criteria, each test result report was annotated into one of seven classes: no PE or DVT, PE only, proximal DVT, distal DVT, thrombophlebitis, other DVT, or both PE and DVT. If the documentation was ambiguous or difficult to interpret, a second expert was consulted and a consensus decision was made. Formal inter-rater agreement statistics were not computed, as dual review was applied only to a limited number of uncertain cases. Among patients confirmed to have VTE, a subset was selected to examine whether their test report findings matched the ICD-10 codes recorded, thereby assessing the consistency between those two data sources.

### Machine learning-based classification with word embedding and active learning

We divided the collected test reports into training and test sets using a computerized randomization method, with an 8:2 ratio of training to test data. Before applying active learning, each test report, written in free-text form, was vectorized via word embedding for efficient processing. In this study, we adopted three word embedding methods—Term Frequency (TF), Word2Vec, and Doc2Vec [24,25]. TF is a bag-of-words approach that converts each word in a document into a unique index and stores the frequency of occurrence at the corresponding index. Word2Vec transforms each word in a document into a high-dimensional vector, placing semantically similar words in nearby positions in the vector space. Doc2Vec represents the entire document as a single vector, reflecting its overall context and structure, thus facilitating classification of semantically similar texts in a broader context.

We selected three machine learning models—RF, SVM, and GBM—to train on the vectorized data. For the SVM, we examined an optimization method based on Stochastic Gradient Descent (SGD), hereafter called SVM with SGD, to improve its learning efficiency on large datasets.

Each model was initially trained on 8 samples before we applied the active learning query strategy. After training on those samples, the models selected the most uncertain (highly needed) data from newly presented samples, requested labeling, and were retrained on the newly labeled data. The seven available query strategies were random sampling, three uncertainty-based sampling methods (least confidence, margin sampling, entropy sampling), Length-Words, Word similarity, and Term Frequency–Inverse Document Frequency (TF-IDF) similarity [26].

Least confidence calculates a model’s uncertainty about the most probable (best sequence) label derived from the posterior probability. For a data sample *x*, uncertainty is typically defined by:


$$\begin{array}{c} \phi^{LC}(x)=\text{1}-P\left(y^{*}|x;\theta \right) \end{array}$$ (1)

where $P(y^{*}|x;\theta )$ is the softmax output indicating how likely it is that *x* belongs to the top-predicted class $y^{*}$. A lower confidence implies a higher level of uncertainty (1 – confidence), suggesting a greater need for labeling and retraining on that data.

Margin sampling assesses the difference between the model’s highest and second-highest class probabilities for a given *x*. A smaller difference implies greater uncertainty because it indicates that the model cannot decisively distinguish between the top two classes ($y_{\text{1}}^{*}$ and $y_{\text{2}}^{*}$). The margin of classification can be represented as:


$$\begin{array}{c} \phi^{MS}(x)=\text{1}-(P\left(y_{\text{1}}^{*}|x;\theta \right)-P\left(y_{\text{2}}^{*}|x;\theta \right)) \end{array}$$ (2)

Entropy sampling measures the disorder (entropy) in the probability distribution across all classes. The more evenly distributed the probabilities, the less confident the model is about any single class, indicating higher uncertainty. The entropy for a data sample is calculated as:


$$\begin{array}{c} \phi^{ES}(x)=-\sum_{c=\text{1}}^{C}P\left(y_{c}|x;\theta \right)\cdot \text{log}P\left(y_{c}|x;\theta \right) \end{array}$$ (3)

Where $P(y_{c}|x;\theta )$ is the probability that sample *x* belongs to class *c*, and $-\text{log}P(y_{c}|x;\theta )$ denotes its information entropy. The maximum entropy occurs when the probability distribution is nearly uniform, signifying that the model cannot confidently assign *x* to any single class.

Length-Words assumes that sentences with more words potentially contain more information than sentences with fewer words. Thus, samples (sentences) with a greater word count are prioritized for training.

Word similarity uses cosine similarity among data points vectorized via word embedding. The data sample that yields the lowest cosine similarity to already-learned samples is considered “most novel,” prompting the model to request labeling to capture new patterns the model has not yet fully learned.

TF-IDF similarity applies an IDF weight to the TF-based embedding, emphasizing rare words. Samples with the lowest cosine similarity are then selected for training. Cosine similarity is defined as:


$$\begin{array}{c} \text{Cosine similarity}=\text{cos}\theta =\frac{A\cdot B}{\left|A||B\right|}=\frac{\sum_{i=\text{1}}^{n}A_{i}B_{i}}{\sqrt{\sum_{i=\text{1}}^{n}{{(A}_{i})}^{\text{2}}\times \sum_{i=\text{1}}^{n}{{(B}_{i})}^{\text{2}}}} \end{array}$$ (4)

When using TF for word embedding, we applied all seven active learning strategies to train and evaluate the machine learning models. However, when Word2Vec or Doc2Vec was used for embeddings, we used Word similarity alone or Word similarity combined with one of three uncertainty-based sampling methods (least confidence, margin sampling, or entropy sampling)—, which was collectively termed “Word similarity uncertainty sampling.” To accomplish this, we computed the similarity of newly presented data *u* to already-labeled data and calculated each class *c*’s average similarity as follows:


$$\begin{array}{c} \text{Average similarity}(\text{Class}c)=\frac{\text{1}}{\left|L_{c}\right|}\sum_{x\in L_{c}}\text{Cosine similarity}(x,u) \end{array}$$ (5)

Here, *L*_*c*_ is the set of labeled data belonging to class *c*, and cosine similarity(*x, u*) is between vectorized data *x* and *u*. We used those average similarities, as we integrated the uncertainty-based sampling (least confidence, margin, entropy) to form the Word similarity uncertainty sampling strategy.

**Fig 1:**
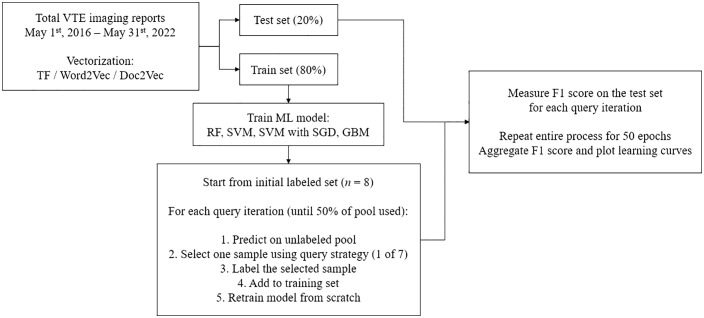
Overview of the active learning workflow for VTE imaging test report classification. The total dataset of imaging reports was split into a training set (80%) and a test set (20%). Three vectorization methods (TF, Word2Vec, Doc2Vec) were applied. Four machine learning classifiers (RF, SVM, SVM with SGD, GBM) were trained using active learning strategy. One epoch started with 8 labeled samples. At each query iteration, the model predicted labels on the unlabeled pool, and one sample was selected for labeling based on one of seven query strategies. The newly labeled sample was added to the training set, and the model was retrained from scratch. This process continued until 50% of the training pool had been labeled. The F1 score was measured on the test set after each query iteration. The entire process was repeated for 50 epochs, and performance metrics were aggregated to plot learning curves.

In this study, we defined *one epoch* as a full active learning cycle starting from a small labeled set of 8 samples. During each epoch, we performed a series of query iterations in which, at every step, the model predicted on the unlabeled pool, selected one sample according to a predefined query strategy, added the newly labeled sample to the training set, and then retrained the model from scratch. After each query iteration, the model’s F1 score was recorded on a fixed test set. This process continued until 50% of the training data had been used, at which point the epoch was complete. To account for randomness in sample selection and model initialization, we repeated this entire process identically for 50 independent epochs. At the end of all repetitions, we aggregated the F1 scores from each corresponding query iteration across all 50 epochs and averaged them. The purpose of this design was to reduce the variance inherent in small-sample active learning and to better characterize the typical performance trajectory of each approach. Fig 1 summarizes the active learning workflow.

### Deep learning-based classification

Next, we evaluated the classification performance of the deep learning models—BERT, 1D-CNNs, and LSTM—on the test reports. Because these reports predominantly contain biomedical terms, the pre-trained BioBERT and BioLinkBERT were also used to see whether they handled domain-specific text more effectively. Moreover, given the varying lengths and textual patterns of the test reports (and the fact that identical results could be expressed differently), we adopted two 1D-CNN variants; a Multi-Kernel 1D-CNN and a Multi-Kernel 1D-CNN with Global Vectors for Word Representation (GloVe). In total, seven models were tested: BERT, BioBERT, BioLinkBERT, 1D-CNN, Multi-Kernel 1D-CNN, Multi-Kernel 1D-CNN with GloVe, and LSTM.

During preprocessing, each radiology report was tokenized using the tokenizer provided with the respective deep learning model (e.g., BERT tokenizer). We set the maximum input length to 256 tokens, where a token refers to the unit used by the tokenizer, typically comprising individual words or subword segments. If the tokenized sequence was shorter than 256 tokens, padding tokens were added; if longer, the excess tokens were truncated from the end. Each model was trained for 50 epochs, where one epoch refers to one complete pass through the training dataset. Additionally, we applied 5-fold cross-validation to maximize the use of available training data while ensuring robustness in performance evaluation.

For training, we used Adaptive Moment Estimation (ADAM) as the optimizer and implemented early stopping to prevent overfitting if validation performance failed to improve. Specifically, if the validation accuracy did not improve by at least 0.0001 for two consecutive epochs, training was halted.

The test reports used in this study were annotated into seven classes, among which the “no DVT or PE” class comprised the largest proportion, raising concerns about potential class imbalance. Accordingly, we investigated performance differences between two scenarios: classifying all seven classes versus classifying only the six smaller classes (excluding the “no DVT and PE” class).

For BERT, BioBERT, and BioLinkBERT, we tuned the hyperparameters to find each model’s optimal settings. Concretely, we tried batch sizes of 32 and 64, and learning rates of 0.00001, 0.00003, and 0.00005, to compare higher learning rates (faster but possibly higher loss) and lower learning rates (slower but potentially minimizing loss). We also evaluated how stemming, lemmatization, and lowercasing affected performance.

### Performance evaluation

In this study, we use the F1 score to assess model performance after each active learning query iteration. The F1 score is defined as the harmonic mean of precision and recall, calculated as follows:


$$\begin{array}{c} \text{Precision}=\frac{\text{True Positive}}{\text{True Positive}+\text{False Positive}} \end{array}$$ (6)

$$\begin{array}{c} \text{Recall}=\frac{\text{True Positive}}{\text{True Positive}+\text{False Negative}} \end{array}$$ (7)

$$\begin{array}{c} \text{F1 score}=\text{2}\cdot \frac{\text{Precision}\cdot \text{Recall}}{\text{Precision}+\text{Recall}} \end{array}$$ (8)

In addition to evaluating classification performance using the F1 score, we also measured the inference time per sample after model training. For deep learning models in particular, inference time was defined as the average time required for a trained model to process a single radiology report and produce a classification output.

### Study ethics

This study was conducted with the approval of the Institutional Review Board (IRB) of Seoul St. Mary’s Hospital. All information that could identify the study subjects was anonymized (IRB# KC22RISI0470). The requirement for consent was waived because this was a retrospective study using de-identified medical records that were fully anonymized prior to data access. The data had been collected during routine clinical care and included no personally identifiable information such as patient names or identification numbers. All data were anonymized by a designated privacy officer before being accessed by the researchers. Given the nature of the study, it was not feasible to obtain consent from each individual without compromising the study’s validity. The IRB determined that the study posed minimal risk to participants and that waiver of consent would not adversely affect their rights or welfare.

## Results

### Datasets of interest

During the data collection period, 5,839 imaging tests were performed at our institution for inpatients suspected of having VTE. Specifically, there were 2,425 cases of chest CT, 69 cases of pulmonary artery CT angiography, 568 cases of DVT CT, 886 cases of lower extremity vein CT, and 1,891 cases of lower extremity vein duplex ultrasonography. Among those patients, 2,953 were male (50.6%) and 2,886 were female (49.4%), with an average age of 61.9 years (Table 1).

**Table 1:** Demographics and Modality of imaging tests undergone by the study participants.

	Statistics (n = 5,839)
***Demographics***	
Sex	
Male	2,953 (50.6%)
Female	2,886 (49.4%)
Age	61.9 (+/- 15.1)
***Modality of imaging test***	
Chest CT	2,425 (41.5%)
Pulmonary artery CT angiography	69 (1.2%)
Lower extremity vein CT	568 (9.7%)
Deep vein thrombosis CT	886 (15.2%)
Lower extremity vein duplex ultrasonography	1,891 (32.4%)

Data are presented as n (%) or mean (+/- standard deviation).

CT, Computed tomography.

Of the 5,839 imaging tests, VTE was detected in 1,088 cases (18.6%). Among the VTE-positive cases, there were 75 cases of PE only (6.9%), 570 cases of proximal DVT (52.4%), 286 cases of distal DVT (26.3%), 58 cases of thrombophlebitis (5.3%), and 11 cases of other DVT (1.0%); additionally, 88 cases (8.1%) involved both DVT and PE (Table 2).

**Table 2:** Prevalence and classification of VTE.

Classification	Statistics (n = 5,839)
***Presence of VTE***	
VTE Present	1,088 (18.6%)
No VTE	4,751 (81.4%)
***Classification of VTE***	
PE only	75 (6.9%)
Proximal DVT	570 (52.4%)
Distal DVT	286 (26.3%)
Thrombophlebitis	58 (5.3%)
Other DVT	11 (1.0%)
DVT and PE	88 (8.1%)

Data are presented as n (%).

VTE, Venous thromboembolism; PE, Pulmonary embolism; DVT, Deep vein thrombosis.

To examine how many patients with VTE-positive test reports actually received ICD-10 codes indicating the VTE. We randomly selected 572 VTE-positive tests and reviewed the assigned ICD-10 codes. We found that 372 (65.0%) carried a VTE-related ICD-10 code (Table 3).

**Table 3:** ICD-10 code matching in a random sample of VTE-positive test reports (n = 572).

ICD-10 Code Status	n (%)
VTE-related ICD-10 code assigned	372 (65.0%)
No VTE-related ICD-10 code assigned	200 (35.0%)
Total	572

ICD, International Classification of Diseases; VTE, Venous thromboembolism.

### Machine learning-based classification of test reports

**Fig 2:**
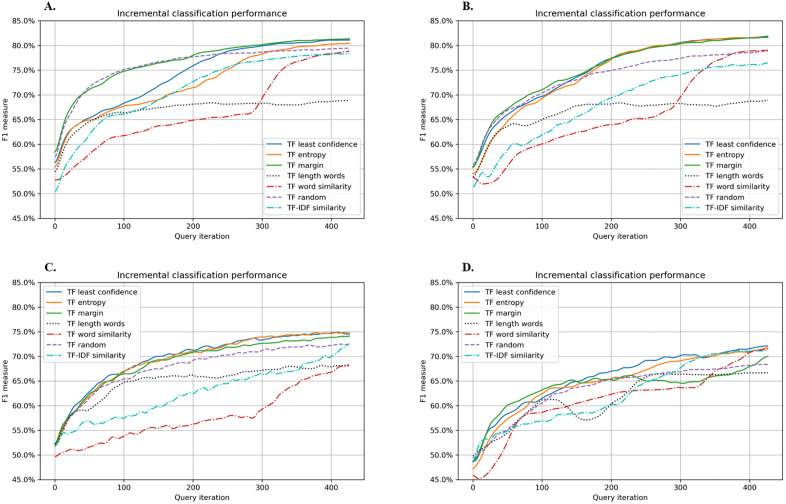
Performance graphs of active learning with the seven query strategies using term frequency for the word embedding. A. Random Forest, B. Gradient Boosting Machine, C. Support Vector Machine, D. Support Vector Machine with Stochastic Gradient Descent.

Fig 2 illustrates the F1 scores achieved by each model across the various query iterations when TF-IDF–based word embedding was applied with the seven active learning strategies. When the RF was trained using random sampling or margin sampling, it attained the highest performance at query iteration 100, with an F1 score of approximately 0.75. Following that, GBM, SVM with SGD, and SVM reached maximum F1 scores of around 0.70, 0.67, and 0.63, respectively. Notably, both the RF and GBM eventually exceeded an F1 score of 0.80, indicating robust classification capability, whereas SVM with SGD and SVM remained at roughly 0.75 and 0.72, respectively. Of particular interest, Word similarity and TF-IDF similarity (both reliant on cosine similarity) tended to yield the lowest performance at query iteration 100.

**Fig 3:**
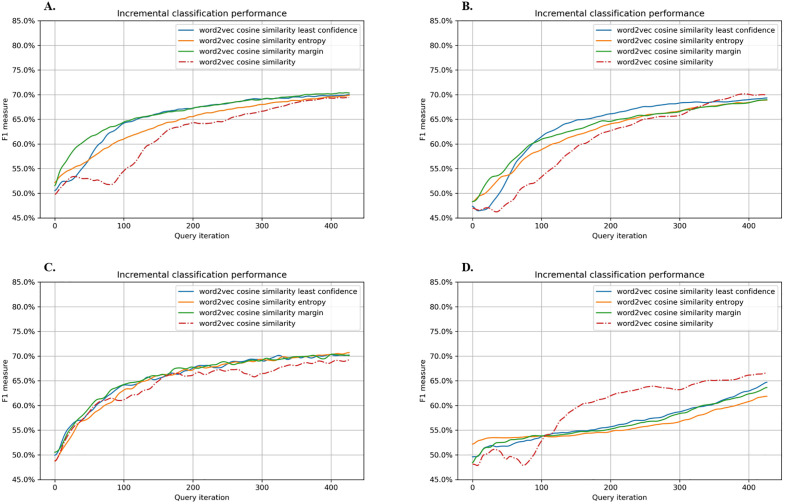
Performance graphs of active learning using Word2Vec for word embedding. The query strategies were either word similarity alone or word similarity combined with least confidence, entropy sampling, or margin sampling. A. Random Forest, B. Gradient Boosting Machine, C. Support Vector Machine, D. Support Vector Machine with Stochastic Gradient Descent.

Fig 3 shows the model performance when Word2Vec was used for word embedding, and Word similarity was combined with least confidence, entropy sampling, or margin sampling. At query iteration 100, RF and SVM with SGD both achieved the highest F1 scores of around 0.65, followed by SVM at roughly 0.62 and GBM below 0.55. Ultimately, RF, SVM, and SVM with SGD converged at F1 scores of about 0.70, with no striking differences among the query strategies. In contrast, GBM performed best (~0.66) when Word similarity alone was applied; incorporating uncertainty-based methods reduced its F1 score to the 0.65–0.60 range.

Overall, the different query strategies produced no major differences in final F1 scores. However, when Word similarity was used alone, certain intervals exhibited a drop in performance during active learning, making prediction less stable. In contrast, coupling Word similarity with an uncertainty-based method resulted in more consistent improvement, culminating in comparatively higher F1 scores at query iteration 100.

**Fig 4:**
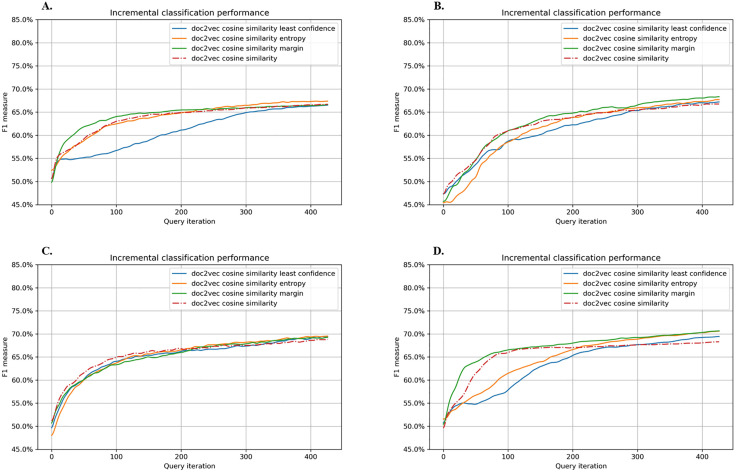
Performance graphs of active learning using Doc2Vec for word embedding. The query strategies were either word similarity alone or word similarity combined with least confidence, entropy sampling, or margin sampling. A. Random Forest, B. Gradient Boosting Machine, C. Support Vector Machine, D. Support Vector Machine with Stochastic Gradient Descent.

Fig 4 shows results using Doc2Vec-based word embedding with the same training procedure used with Word2Vec. RF, SVM, and SVM with SGD all reached their highest F1 scores (0.64–0.66) at query iteration 100. Although GBM started lower at around 0.61 at query iteration 100, all models eventually converged within the 0.70–0.65 range, indicating no significant differences across models or query strategies. The instability of Word similarity observed with Word2Vec was not as pronounced with Doc2Vec. During active learning, RF showed slightly reduced performance when coupling cosine similarity with margin sampling, and SVM declined under cosine similarity with entropy or margin sampling. However, those gaps diminished throughout successive training, leading to similar final outcomes.

### Performance of deep learning-based classification

We tested seven deep learning strategies—BERT, BioBERT, BioLinkBERT, 1D-CNN, Multi-Kernel 1D-CNN, Multi-Kernel 1D-CNN with GloVe, and LSTM—to classify the test reports and evaluate their performance. We first examined seven classes: “PE only,” “Proximal DVT,” “Distal DVT,” “Thrombophlebitis,” “Other DVT,” “Both DVT and PE,” and “No DVT or PE.” LSTM achieved the highest F1 score (0.9513), followed by the Multi-Kernel 1D-CNN with GloVe at 0.9492. Differences among the other models were not substantial, and all except 1D-CNN surpassed an F1 score of 0.94.

These findings likely stem from the severe class imbalance, with more than 80% of the training data labeled “No DVT or PE.” We therefore removed that class and re-evaluated the remaining six classes. In this 6-class scenario, LSTM again attained the highest F1 score (0.9168), followed by the Multi-Kernel 1D-CNN with GloVe (0.9093). However, those score represent a sizeable drop from the 7-class scores. In fact, all models showed a notable decrease in F1 score in the 6-class scenario. BioBERT, in particular, declined by about 0.09. Table 4 summarizes the 6-class and 7-class classification outcomes using the seven strategies.

**Table 4:** Performance of the deep learning models in classifying pulmonary embolism and venous thromboembolism. 6-Class classification excludes the “No DVT and PE” class.

	BERT	BioBERT	BioLinkBERT	1D CNN	Multi-kernel 1D CNN	Multi-kernel 1D CNN with GloVe	LSTM
**6-Class**	0.8631	0.8576	0.8815	0.8493	0.8943	0.9093	0.9168
**7-Class**	0.9435	0.9474	0.9490	0.9322	0.9449	0.9492	0.9513

PE, Pulmonary embolism; DVT, Deep vein thrombosis; BERT, Bidirectional Encoder Representations from Transformers; 1D CNN, one-dimensional convolutional neural networks; LSTM, Long Short-Term Memory.

For the 6-class setting, the best-performing BERT model was the BERT-base-multilingual-cased with a batch size of 64, a learning rate of 0.00003, and no stemming, lemmatization, or lowercasing. BioBERT (BioBERT-base-cased-v1.2) achieved its highest F1 score at a batch size of 32, a learning rate of 0.00005, and similarly no stemming, lemmatization, or lowercasing. By contrast, BioLinkBERT (BioLinkBERT-base) performed best under a batch size of 32, a learning rate of 0.00003, and full preprocessing (stemming, lemmatization, and lowercasing). S1 and S2 Tables detail the various hyperparameter combinations tested for BERT, BioBERT, and BioLinkBERT, along with their corresponding F1 scores.

In the 6-class classification task, we measured the average inference time of each deep learning model using a batch size of 1. For transformer-based models, the average inference time per report was 0.0238 second for BERT, 0.0238 second for BioBERT, and 0.0240 second for BioLinkBERT (based on 217 test samples). For CNN-based models and LSTM, the inference time was significantly lower: 0.00180 second for 1D-CNN, 0.00154 seconds for multi-kernel 1D-CNN, 0.00149 seconds for multi-kernel 1D-CNN with GloVe, and 0.00146 seconds for LSTM (based on 71 test samples). All timings include both tokenization and classification steps.

## Discussion

VTE can be confirmed via CT or ultrasound, and results are often documented in free-text format. Although these reports can be used to identify VTE in inpatients, previous studies have pointed out that manually assigning ICD-10 codes might underestimate the actual prevalence of VTE [9]. In line with those findings, only 65.0% of the 1,088 VTE cases confirmed in this study had corresponding ICD-10 diagnostic codes in their records.

Analyzing the original imaging reports instead of relying on ICD-10 codes could be a promising way to accurately estimate VTE prevalence. However, having experienced specialists annotate every report is both time- and labor-intensive, underscoring the need for an efficient approach. For example, active learning for clinical text classification is recognized for achieving performance comparable to random sampling [16,17,26]. Therefore, we investigated whether active learning could classify VTE-related imaging reports more efficiently than random sampling, and, leveraged natural language processing (NLP), to see whether we could achieve high accuracy with minimal training data.

We compared various word embedding techniques and query strategies and found that using word similarity alone produced low or unstable performance in the early training phase (query iteration ~100). Although performance improved and stabilized during successive iterations, it fell short of the initial goal of “achieving high accuracy with minimal training.” In contrast, with Word2Vec-based embedding, combining word similarity with an uncertainty-based approach facilitated relatively faster stabilization, though Doc2Vec (another dense representation) did not exhibit the same pattern. Generally, embeddings using dense representations (Word2Vec, Doc2Vec) tended to underperform embeddings using sparse representations (TF).

Although the overall F1 scores of the machine learning–based models ranged from 0.70 to 0.80, most models demonstrated a plateau in performance toward the end of training. Despite the moderate dataset size, the classification accuracy of traditional machine learning models remained limited, and further gains are unlikely to result simply from increasing the amount of labeled data. This limitation is expected to be exacerbated in future multi-institutional settings due to increased data heterogeneity. Accordingly, machine learning–based approaches may struggle to deliver clinically meaningful performance in more complex or diverse environments. Future model development and validation efforts should therefore focus on deep learning–based models, which demonstrated consistently higher performance across all evaluation settings in this study.

Therefore, we also used deep learning models to classify each imaging test report to see whether those results would be more precise. We found that LSTM attained an F1 score above 0.95 in the 7-class scenario, and the BioBERT and BioLinkBERT performed slightly better than the naive BERT. Nevertheless, BioLinkBERT was not substantially superior to the 1D-CNN or LSTM, and in some cases performed comparably or slightly worse.

BERT-based models are generally considered to be more accurate than 1D-CNN or LSTM. Their comparatively lower performance in this study can be attributed to the limited size of the dataset. BERT models typically require large-scale data and mandatory fine-tuning to reach their full potential. Even so, we maximized the BERT performance by tuning the hyperparameters, which raised the 6-class classification score from a minimum of 0.7785 to 0.8630 and improved the 7-class classification from 0.9070 to 0.9435. With further data collection and continued hyperparameter optimization, it might be possible to annotate test reports at reduced cost while more accurately determining the actual prevalence of VTE.

Additionally, inference time analysis showed that all deep learning models produced classification results within a clinically acceptable timeframe. BERT-based models required approximately 0.023–0.024 seconds per report, which may be relatively slow but still allows for practical deployment in non-urgent clinical settings. In contrast, LSTM and CNN-based models demonstrated significantly faster inference times (around 0.0014–0.0018 seconds per report), making them particularly suitable for semi-automated review systems or potential integration into routine workflows. Given the moderate complexity of the classification task in this study, deploying lighter-weight models such as LSTM or multi-kernel CNN may offer a more favorable trade-off between speed and accuracy than heavier transformer-based architectures. However, as our findings are based on single-center data, further validation across institutions is essential to confirm whether this observation holds in more diverse clinical environments.

This study has several limitations. First, we aimed to determine whether deep learning models which typically require large-scale labeled datasets and high computational resources could achieve strong classification performance when trained on relatively small amounts of data using active learning. This approach was motivated by the practical constraints of manual annotation and the computational burden associated with large language models. However, relying on data from a single institution raises concerns about generalizability. Radiology reports often differ in structure, terminology, and reporting practices across institutions, which may limit the external validity of our findings. Although external validation was not feasible within the scope of the current study due to data access limitations, we acknowledge that such validation is crucial.

To address this, future work could adopt a sequential validation strategy in which data from additional institutions are incrementally incorporated into training and validation. This would allow for a more realistic assessment of how well the model generalizes across diverse clinical environments and could help identify domain-specific language patterns that hinder extrapolation. In addition, as the size and diversity of data increase, methods such as knowledge distillation or model pruning may help reduce computational burden while preserving model performance in deployment settings.

Second, because more than 80% of the tests were negative for VTE and certain classes (e.g., distal DVT) constituted only about 1% of the positive cases, severe class imbalance was evident. One strategy to address class imbalance is undersampling, where an equal number of samples are randomly selected from each class based on the size of the least frequent class, thereby achieving a uniform class distribution during training. However, in our dataset, the “Other DVT” class included only 11 samples, accounting for just 1.0% of the VTE-present cases. Adjusting all other classes to match this minimal count would substantially reduce the usable training data and likely impair model generalizability. This limitation stems from an inherent clinical reality, as such rare phenotypes are naturally underrepresented in real-world hospital settings. Addressing this issue requires the acquisition of additional minor-class data or the use of stratified sampling to maintain balanced training/test sets. However, even multi-center data might not guarantee sufficient samples for the minority classes, so data augmentation or methods such as the synthetic minority over-sampling technique (SMOTE), followed by stratified sampling, are needed to improve model balance.

Third, even after training on multi-center data, prediction errors might persist. To ensure that clinicians can trust and act upon model outputs, techniques that explain each model’s contextual reasoning are required. Thus, methods such as Local Interpretable Model-Agnostic Explanations (LIME) or Shapley Additive Explanations (SHAP) should be considered to enhance interpretability and clinician confidence. These methods are designed to explain the predictions of complex models such as deep neural networks. They do so by estimating how much each word or phrase in the input text contributes to the final prediction. For instance, in a report containing the phrase “no evidence of pulmonary embolism”, such interpretability tools could help verify that the model appropriately increases the probability of predicting “No PE or DVT” class in response to this negating phrase. These explanations would provide insights into the model’s token-level or phrase-level contributions to its predictions, helping clinicians better understand and validate the reasoning process. Although not implemented in the current study, we plan to incorporate such analyses in future work to improve transparency and foster clinical acceptance.

Fourth, to evaluate the model in real clinical settings, prospective studies are needed, alongside external validation, to verify that the performance observed with the original dataset can be maintained in real-world conditions.

In summary, although we attempted to classify VTE imaging reports with minimal labeled data by combining machine learning models with active learning, our initial objective of achieving robust performance with limited training was not fully realized. Model performance required a certain amount of training to stabilize, and minority classes (e.g., distal DVT) were not sufficiently learned, resulting in fluctuating performance. As a result, active learning alone was insufficient to reach clinically required accuracy. On the other hand, applying deep learning models—particularly LSTM and Multi-Kernel 1D-CNN with GloVe—yielded higher accuracy, and the BERT-based models showed potential for improvement if more data or further hyperparameter optimization become available. Still, the high computational cost of deep learning suggests that future work should use techniques such as knowledge distillation and pruning to reduce model size and inference time, thereby enhancing the feasibility of real clinical deployment.

## Conclusion

This study compared machine learning and deep learning methods for automated VTE imaging report classification, incorporating active learning to reduce labeling costs. Combining machine learning and active learning produced instability in the early training phase and did not always achieve sufficiently high average accuracy for clinical application, through more diverse metrics might be needed to evaluate the final performance. On the other hand, deep learning models (e.g., LSTM, Multi-Kernel 1D-CNN with GloVe) attained relatively high accuracy, and BERT-based models showed potential for improvements with additional data or hyperparameter tuning. However, the high computational expense of deep learning remains a challenge. Future research should apply strategies such as knowledge distillation or pruning to enhance computational efficiency, address class imbalance, and improve model interpretability, thereby paving the way for practical clinical adoption of automated VTE report classification.

## Supporting information

S1 Table Classification performance of BERT, BioBERT, BioLinkBERT using 6 classes and various experimental settings. BERT, Bidirectional Encoder Representations from Transformers.
(DOCX)

S2 Table Classification performance of BERT, BioBERT, BioLinkBERT using 7 classes and various experimental settings. BERT, Bidirectional Encoder Representations from Transformers.
(DOCX)
