# Supplementary material for: Detection and classification of venous thromboembolism through image test reports analysis using active learning and deep learning
Source: PLoS One. 2025 Nov 10;20(11):e0335262. doi: 10.1371/journal.pone.0335262 (PMC12599933; doi:10.1371/journal.pone.0335262)
Supplement: S1 Table — BERT, Bidirectional Encoder Representations from Transformers. (DOCX) [file pone.0335262.s001.docx]

**Supplementary Table 1**. Classification performance of BERT, BioBERT, BioLinkBERT using 6 classes and various experimental settings.

| Model | Max length | Batch size | Learning rate | F1 score | Preprocessing |
| --- | --- | --- | --- | --- | --- |
| BERT-base-uncased | 256 | 64 | 5.E-05 | 0.79504 |  |
| BERT-base-uncased | 256 | 64 | 3.E-05 | 0.79594 |  |
| BERT-base-uncased | 256 | 64 | 1.E-05 | 0.79323 |  |
| BERT-base-uncased | 256 | 32 | 5.E-05 | 0.78584 |  |
| BERT-base-uncased | 256 | 32 | 3.E-05 | 0.79686 |  |
| BERT-base-uncased | 256 | 32 | 1.E-05 | 0.79318 |  |
| BERT-base-multilingual-cased | 256 | 64 | 5.E-05 | 0.85846 |  |
| BERT-base-multilingual-cased | 256 | 64 | 3.E-05 | **0.86306** |  |
| BERT-base-multilingual-cased | 256 | 64 | 1.E-05 | 0.81983 |  |
| BERT-base-multilingual-cased | 256 | 64 | 5.E-05 | 0.75552 | Removing numbers |
| BERT-base-multilingual-cased | 256 | 64 | 3.E-05 | 0.80329 | Removing numbers |
| BERT-base-multilingual-cased | 256 | 64 | 1.E-05 | 0.77848 | Removing numbers |
| BERT-base-cased | 256 | 64 | 5.E-05 | 0.81893 |  |
| BERT-base-cased | 256 | 64 | 3.E-05 | 0.84374 |  |
| BERT-base-cased | 256 | 64 | 1.E-05 | 0.83092 |  |
| BERT-base-cased | 256 | 64 | 5.E-05 | 0.81985 | Removing numbers |
| BERT-base-cased | 256 | 64 | 3.E-05 | 0.82723 | Removing numbers |
| BERT-base-cased | 256 | 64 | 1.E-05 | 0.83457 | Removing numbers |
| BERT-base-cased | 256 | 32 | 5.E-05 | 0.84287 |  |
| BERT-base-cased | 256 | 32 | 3.E-05 | 0.83824 |  |
| BERT-base-cased | 256 | 32 | 1.E-05 | 0.83732 |  |
| BioBERT-base-cased-v1.2 | 256 | 32 | 5.E-05 | **0.85757** |  |
| BioBERT-base-cased-v1.2 | 256 | 32 | 3.E-05 | 0.83003 |  |
| BioBERT-base-cased-v1.2 | 256 | 32 | 1.E-05 | 0.84470 |  |
| BioLinkBERT-base | 256 | 64 | 5.E-05 | 0.80612 |  |
| BioLinkBERT-base | 256 | 64 | 3.E-05 | 0.81341 |  |
| BioLinkBERT-base | 256 | 64 | 1.E-05 | 0.78859 |  |
| BioLinkBERT-base | 256 | 64 | 5.E-05 | 0.79411 | Removing numbers |
| BioLinkBERT-base | 256 | 64 | 3.E-05 | 0.81343 | Removing numbers |
| BioLinkBERT-base | 256 | 16 | 5.E-05 | 0.74459 |  |
| BioLinkBERT-base | 256 | 16 | 3.E-05 | 0.80052 |  |
| BioLinkBERT-base | 256 | 16 | 1.E-05 | 0.80701 |  |
| BioLinkBERT-base | 256 | 32 | 3.E-05 | 0.87320 | Stemming and lemmatization |
| BioLinkBERT-base | 256 | 32 | 3.E-05 | **0.88145** | Stemming, lemmatization, lowercasing and removing numbers |

BERT, Bidirectional Encoder Representations from Transformers.
