# Supplementary material for: Detection and classification of venous thromboembolism through image test reports analysis using active learning and deep learning
Source: PLoS One. 2025 Nov 10;20(11):e0335262. doi: 10.1371/journal.pone.0335262 (PMC12599933; doi:10.1371/journal.pone.0335262)
Supplement: S2 Table — BERT, Bidirectional Encoder Representations from Transformers. (DOCX) [file pone.0335262.s002.docx]

**Supplementary Table 2**. Classification performance of BERT, BioBERT, BioLinkBERT using 7 classes and various experimental settings.

| Model | Max length | Batch size | Learning_rate | F1 score | Preprocessing |
| --- | --- | --- | --- | --- | --- |
| BERT-base-multilingual-cased | 256 | 64 | 5.00E-05 | 0.90701 |  |
| BERT-base-multilingual-cased | 256 | 64 | 3.00E-05 | 0.93218 |  |
| BERT-base-multilingual-cased | 256 | 64 | 1.00E-05 | 0.92499 |  |
| BERT-base-multilingual-cased | 256 | 32 | 5.00E-05 | 0.92122 |  |
| BERT-base-multilingual-cased | 256 | 32 | 3.00E-05 | 0.93732 |  |
| BERT-base-multilingual-cased | 256 | 32 | 3.00E-05 | 0.92704 | Stemming, lemmatization and lowercasing |
| BERT-base-cased | 256 | 64 | 5.00E-05 | 0.92208 |  |
| BERT-base-cased | 256 | 64 | 3.00E-05 | 0.92927 |  |
| BERT-base-cased | 256 | 64 | 1.00E-05 | 0.92807 |  |
| BioBERT-base-cased-v1.2 | 256 | 64 | 5.00E-05 | 0.91043 |  |
| BioBERT-base-cased-v1.2 | 256 | 64 | 3.00E-05 | 0.93663 |  |
| BioBERT-base-cased-v1.2 | 256 | 64 | 1.00E-05 | 0.92619 |  |
| BioBERT-base-cased-v1.2 | 256 | 32 | 3.00E-05 | 0.94554 | Stemming and lowercasing |
| BioBERT-base-cased-v1.2 | 256 | 32 | 5.00E-05 | 0.94246 | Stemming and lowercasing |
| BioBERT-base-cased-v1.2 | 256 | 32 | 1.00E-05 | 0.94451 | Stemming and lowercasing |
| BioBERT-base-cased-v1.2 | 256 | 32 | 5.00E-05 | 0.94177 |  |
| BioBERT-base-cased-v1.2 | 256 | 32 | 3.00E-05 | 0.94622 |  |
| BioBERT-base-cased-v1.2 | 256 | 32 | 1.00E-05 | 0.94434 |  |
| BioLinkBERT-base | 256 | 64 | 5.00E-05 | 0.92413 |  |
| BioLinkBERT-base | 256 | 64 | 3.00E-05 | 0.92327 |  |
| BioLinkBERT-base | 256 | 64 | 1.00E-05 | 0.92858 |  |
| BioLinkBERT-base | 256 | 32 | 3.00E-05 | 0.93937 | Stemming, lemmatization and lowercasing |
| BioLinkBERT-base | 256 | 32 | 5.00E-05 | 0.92789 | Stemming, lemmatization and lowercasing |
| BioLinkBERT-base | 256 | 32 | 1.00E-05 | **0.94777** | Stemming, lemmatization and lowercasing |
| BioLinkBERT-base | 256 | 16 | 1.00E-05 | 0.94554 | Stemming, lemmatization and lowercasing |
| BioLinkBERT-base | 256 | 32 | 1.00E-05 | 0.94417 | Stemming, lemmatization and lowercasing |
| BioLinkBERT-base | 256 | 32 | 1.00E-05 | 0.94040 | Stemming and lowercasing |

BERT, Bidirectional Encoder Representations from Transformers.
